# Latent viruses can cause disease by disrupting the competition for the limiting factor p300/CBP

**DOI:** 10.1186/s11658-018-0121-1

**Published:** 2018-11-26

**Authors:** Hanan Polansky, Hava Schwab

**Affiliations:** The Center for the Biology of Chronic Disease (CBCD), 616 Corporate Way, Suite 2-3665, Valley Cottage, New York City, NY 10989 USA

**Keywords:** δEF1, MYOD, HIF1α, p53, CBP, p300, Limiting, Microcompetition, Transcription factor

## Abstract

CBP and p300 are histone acetyltransferase coactivators that control the transcription of numerous genes in humans, viruses, and other organisms. Although two separate genes encode CBP and p300, they share a 61% sequence identity, and they are often mentioned together as p300/CBP. Zhou et al. showed that under hypoxic conditions, HIF1α and the tumor suppressor p53 compete for binding to the limiting p300/CBP coactivator. Jethanandani & Kramer showed that δEF1 and MYOD genes compete for the limited amount of p300/CBP in the cell. Bhattacharyya et al. showed that the limiting availability of p300/CBP in the cell serves as a checkpoint for HIF1α activity. Here, we use the microcompetition model to explain how latent viruses with a specific viral cis-regulatory element in their promoter/enhancer can disrupt this competition, causing diseases such as cancer, diabetes, atherosclerosis, and obesity.

With at least 315 different cellular and viral interacting proteins, CBP and p300 are considered the most heavily connected coactivators in the mammalian protein–protein interaction network [1, 2]. Both are histone acetyltransferases, and they control the transcription of numerous genes in humans, viruses, and other organisms. Although two separate genes encode CBP and p300, they share a 61% sequence identity, and they are often mentioned together as p300/CBP [3].

p300/CBP is a 300-kDa protein that has a CH2 domain, which contains its acetyltransferase activity, and five protein-binding domains [3]. Many studies have shown that competition for the limiting p300/CBP is an important mechanism that regulates transcription and cellular activities. This commentary discusses three of these studies [4–6] and connects their observations to the microcompetition model [7, 8].

Using differential equations and a dimensionless state variable, Zhou et al. [5] determined the effect of p300 on the steady-state concentrations of proteins. They discovered that under hypoxic conditions, HIF1α and the tumor suppressor p53 compete for binding to the coactivator p300. They showed that p300 is required for full transcriptional activity of both p53 and HIF1α. According to Zhou et al., this competition indicates that p300 is limiting.

The α7 integrin is involved in the differentiation of myoblasts and is negatively regulated by δEF1, a zinc finger transcription factor, and positively regulated by MYOD. δEF1 has an NR (negative region) domain that binds the p300/CBP coactivator. Overexpression of δEF1 inhibits muscle cell differentiation and represses the activation of the muscle creatine kinase enhancer. On the other hand, MYOD activates muscle genes by binding p300, and uses p300/CBP histone acetylase activity to allow for transcription [4].

Jethanandani & Kramer [4] transfected C2C12 cells with the p400 fragment of the α7 integrin promoter. Then, they co-transfected the cells with either δEF1 alone, δEF1 and MYOD, or δEF1 and CBP, and measured the CAT reporter activity. They observed that CBP increased CAT activity, i.e., an increase in CBP levels mitigated the repression of α7 by δEF1. Based on their results, Jethanandani & Kramer concluded that p300/CBP is limiting, and that δEF1 competes with MYOD for the limited amounts of p300/CBP in the cell.

Bhattacharyya et al. [6] infected human gastric epithelium cells with *Helicobacter pylori*. The results showed an increase in transcription complex formation at the HREs (hypoxia-response elements) of the mcl1 promoter. Then, they observed that the complex included p300, HIF1α, and APE1 (apurinic/apyrimidinic endonuclease 1). Western blotting on whole cell lysates from AGS cells showed that the binding of p300 to the hif1α promoter decreased at higher levels of *H. pylori* infection, without a decrease in the p300 concentration. Moreover, they found that higher levels of *H. pylori* infection increased the expression of hif1α, but decreased the expression of the mcl1 promoter, which is transactivated by HIFα. They discovered that at higher *H. pylori* levels, HIF1α binds to the HIF-binding site (HBS) on the hif1α promoter. Since the HBS is transcriptionally inactive (it lacks the required HIF ancillary sequence, denoted as HAS), this binding does not further transactivate the hif1α promoter. However, this binding has a sequestering effect that limits the intracellular availability of the HIF1α•p300 complex to the mcl1 gene, which decreases mcl1 expression.

The observations in the Bhattacharyya et al. study indicate that p300 is limiting, meaning the HIF1α•p300 complex is limiting. They also show that the decrease in HIF1α•p300 binding to the mcl1 promoter, which decreases mcl1 transcription, is due to competition for the limiting HIF1α•p300 by the hif1α promoter itself. Based on their observations, Bhattacharyya et al. concluded that the limiting availability of p300 in the cell is a checkpoint for HIF1α activity.

These studies showed that competition between cellular transcription factors to bind the limiting p300/CBP is an important regulator of transcription. According to the microcompetition model, disrupting this regulation causes many diseases. The microcompetition model was first described in the book *Microcompetition with Foreign DNA and the Origin of Chronic Disease* [7, 8]. It centers on one type of disruption of this regulation: the one caused by the viruses with an N-box, which is a strong cis-regulatory element found on their promoters/enhancers. During the latent phase, this element binds the cellular GABP•p300/CBP transcription complex, which is limiting, because p300/CBP is limiting. Therefore, the viral N-boxes decrease the availability of GABP•p300/CBP in the cell. The result is abnormal expression of the cellular genes that bind GABP•p300/CBP. The genes that are transactivated by the GABP•p300/CBP complex synthesize fewer proteins, while those that are transrepressed by the complex synthesize more proteins. The abnormal levels of these cellular proteins can cause disease, such as cancer, diabetes, atherosclerosis, and obesity. The book lists human genes that bind the GABP•p300/CBP complex (Table 1) and presents supporting evidence to show that these genes express abnormal levels of their proteins in these diseases.

**Table 1:** List of some human genes that bind the GABP•p300/CBP transcription complex

Gene	Reference
β2 leukocyte integrin (CD18)	Rosmarin et al. 1998 [22]
Interleukin 16 (IL-16)	Bannert et al. 1999 [23]
Interleukin 2 (IL-2)	Avots et al. 1997 [24]
Interleukin 2 receptor β-chain (IL-2Rβ)	Lin et al. 1993 [25]
IL-2 receptor γ-chain (IL-2 γc)	Markiewicz et al. 1996 [26]
Human secretory interleukin-1 receptor antagonist (secretory IL-1ra)	Smith et al. 1998 [27]
Retinoblastoma (Rb)	Sowa et al. 1997 [28]
Human thrombopoietin (TPO)	Kamura et al. 1997 [29]
Aldose reductase	Wang et al. 1993 [30]
Neutrophil elastase (NE)	Nuchprayoon et al. 1999 [31], Nuchprayoon et al. 1997 [32]
Folate binding protein (FBP)	Sadasivan et al. 1994 [33]
Cytochrome c oxidase subunit Vb (COXVb)	Basu et al. 1993 [34], Sucharov et al. 1995 [35]
Cytochrome c oxidase subunit IV	Carter et al. 1994 [36], Carter et al. 1992 [37]
Mitochondrial transcription factor A (mtTFA)	Virbasius et al. 1994 [38]
β subunit of the FoF1 ATP synthase (ATPsynβ)	Villena et al. 1998 [39]
Prolactin (PRL)	Ouyang et al. 1996 [40]
Oxytocin receptor (OTR)	Hoare et al. 1999 [41]

Some common viruses with an N-box are the cytomegalovirus (CMV), Epstein-Barr virus (EBV), herpes simplex virus 1 (HSV-1), human T-cell lymphotropic virus (HTLV), and human immunodeficiency virus (HIV). These viruses are highly prevalent. For instance, an estimated 3.7 billion people worldwide, or around 67% of the global population, are infected with HSV-1 [9]. Since the virus is highly prevalent, why do only a fraction of infected people develop the disease? The answer is that the copy number during latency matters. Only a high enough copy number produces the strong enough sequestering effect that causes a disease (Fig. 1).

**Fig. 1:**
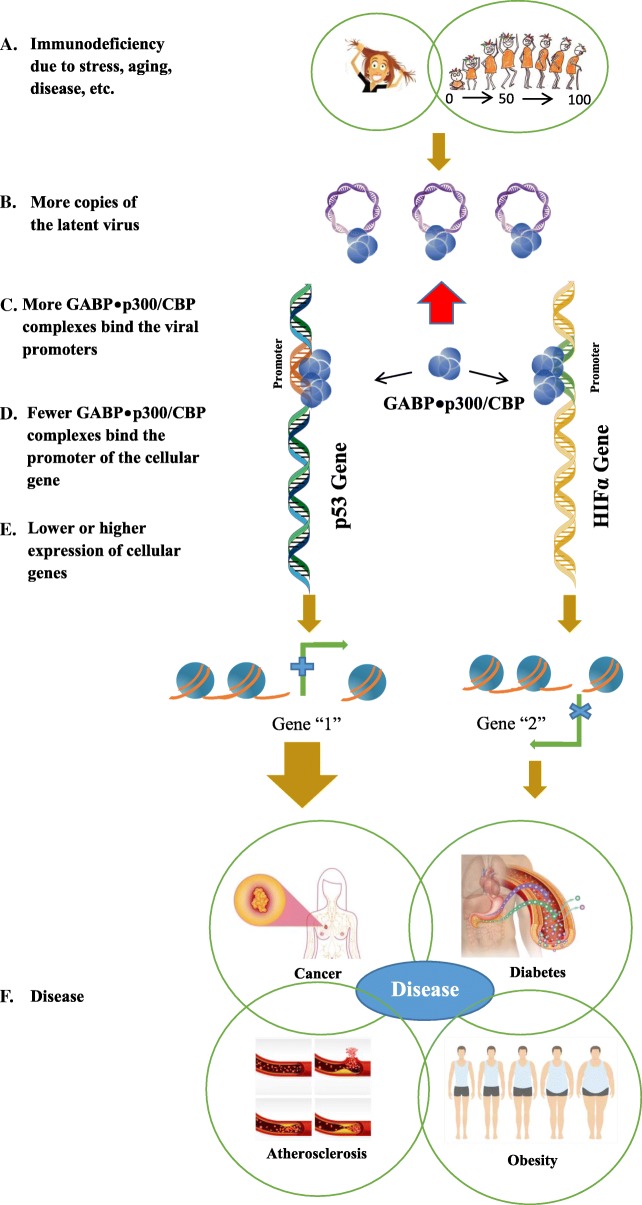
Latent viruses, the microcompetition model, and disease. **a** Immunodeficiency occurs due to stress, aging, disease, etc. **b** More copies of the latent virus are present in the system **c** Due to the increase in the copies of latent viruses, more GABP•p300/CBP complexes bind the viral promoters. **d** As a result, fewer GABP•p300/CBP complexes are available to bind the promoter of the cellular gene. **e** A lower or higher expression of cellular genes. **f** Disease occurs

A recent paper by Zuo et al. [10] showed that the copy number of latent EBV, a virus that binds GABP•p300/CBP during latency, is strongly associated with oncogenicity [11, 12]. What determines the copy number of a virus during its latent phase? It is well known that there is a balance between the efficiency of the immune system and the copy number of latent viruses. It is also known that many events can cause immunodeficiency, including aging [13], certain medications [14, 15], surgery [16–18], chemotherapy [19], radiation [20], and stress [21]. Such events decrease the efficiency of the immune system, increasing the copy number of latent viruses and the risk of disease, as observed by Zuo et al., who stated:

“It has been noticed that EBV load in tumor tissues or blood is associated with the clinical progression and prognosis in both lymphoma and NPC. Our result verifies this association. We also emphasize the importance to measure the level of gene expression or copy number in the virus study instead of only concerning ‘with and without’.”

Our interpretation of the microcompetition model agrees with that of Zuo et al. It is the copy number of the viruses that sequester the limiting GABP•p300/CBP transcription complex and not the ‘infected or not infected’ that determines the fate of the infected individual. Therefore, it should be measured in clinical practice.

To conclude, the microcompetition model explains how an increase in the copy number of a latent virus that binds the limiting GABP•p300/CBP transcription complex increases the sequestering of the complex. This disrupts the allocation of the complex to cellular genes that compete to bind the complex. When this disruption is large enough, the host develops a disease.
